# Alcohol consumption trajectories and risk of breast cancer among postmenopausal women: a Danish cohort study

**DOI:** 10.1007/s10654-024-01179-5

**Published:** 2024-12-04

**Authors:** Christian S. Antoniussen, Cécile Proust-Lima, Daniel B. Ibsen, Anja Olsen, Kim Overvad, Anne Tjønneland, Pietro Ferrari, Christina C. Dahm

**Affiliations:** 1 Department of Public Health, Aarhus University, Aarhus, Denmark; 2 University of Bordeaux, Inserm, Bordeaux Population Health Research Center, Bordeaux, France; 3 Steno Diabetes Center Aarhus, Aarhus University Hospital, Aarhus, Denmark; 4 Department of Nutrition, Exercise and Sports, University of Copenhagen, Copenhagen, Denmark; 5 Danish Cancer Institute, Copenhagen, Denmark; 6 Nutrition and Metabolism Branch, International Agency for Research on Cancer (IARC), World Health Organization, Lyon, France

**Keywords:** Breast cancer, Alcohol consumption, Latent class mixed models, Diet, Cancer, Health cohort

## Abstract

Alcohol consumption is a risk factor for breast cancer (BC), yet little is known about longitudinal alcohol consumption patterns and risk of BC. This study aimed to investigate whether trajectory profiles of alcohol consumption across adulthood were associated with risk of first primary malignant BC in postmenopausal women. At baseline, 28,720 pre-and postmenopausal women aged 50–65 years from the Danish Diet, Cancer and Health Cohort reported their average alcohol intake over the past 12 months and their average alcohol intake at the ages of 20, 30, 40, and 50 years. Alcohol consumption trajectories were estimated using latent class mixed models. BC cases were identified through record linkage to the Danish Cancer Registry. To examine associations between alcohol consumption trajectories and BC, we fitted a proportional hazard model adjusted for potential confounding factors using data from 24,543 postmenopausal women without missing covariate information. We identified 4 alcohol consumption trajectory profiles. During a median follow-up of 16.5 years, 1,591 cases of BC occurred. A mean alcohol consumption trajectory of > 10 g/day was associated with higher risk of BC (HR: 1.65, 95%CI: 1.35–2.03) compared to a mean alcohol consumption trajectory of < 6 g/day. We found no association between trajectory profiles characterized by lower alcohol intakes in early adulthood followed by increasing consumption of alcohol in adulthood compared to a consistently low intake of alcohol. Postmenopausal women drinking consistently high amounts of alcohol throughout adulthood had a higher risk of BC compared to women with a consistently low intake of alcohol.

**Supplementary Information:**

The online version contains supplementary material available at 10.1007/s10654-024-01179-5.

## Introduction

According to the latest GLOBOCAN estimates of cancer incidence and mortality, female breast cancer (BC) accounted for more than 2.0 million incident cases in 2022 [1], making it the second most diagnosed cancer disease with the incidence rate of BC currently increasing among younger women (< 50 years) in many parts of the world [2]. Consumption of alcoholic beverages is an established risk factor for BC, and alcohol consumption was in 2020 estimated to account for 98.300 new BC cases globally (95% uncertainty intervals: 68.200,130.500) [3–5]. However, the established evidence associating alcohol consumption with risk of BC is almost exclusively based on studies considering only single measures of the women’s alcohol intake [6]. A limitation to such approaches is the inability to capture variations in drinking behavior over the course of adulthood which may play a role in relation to future disease risk. In a UK based study focusing on life course alcohol consumption trajectories using data from 9 cohorts with at least three measurement of the participants’ alcohol intake including approximately 59,400men and women, mean consumption of alcohol among women increased considerably during adolescence followed by stable intake in mid-adulthood and a continuous declining intake throughout late-adulthood [7]. As alcohol intake changes over time, it is important to capture variations in alcohol intake when investigating associations between alcohol consumption and risk of various health outcomes. Alcohol intake patterns among women vary not only over time but also between cultures. To date, associations between alcohol trajectories during adulthood and risk of BC have been investigated in an Australian prospective cohort study and in a Spanish population-based case-control study [8, 9]. While these studies represent different alcohol consumption cultures, more research in different populations and time periods is needed to gain insight into the potential importance of consumption patterns over time for BC risk. Additionally, both studies employed group-based trajectory models (GBTM) to identify patterns of alcohol consumption over time [8, 9]. A GBTM is a finite mixture model commonly used to identify subgroups of participants characterized by similar exposure profiles and to summarize variability of predictors over time in longitudinal studies [10]. However, the model may not take the intra-individual correlation between multiple alcohol records from the same woman into account [11–13]. In contrast, latent class mixed models (LCMM) combine GBTM with mixed models to account for intra-individual correlation, and were recently used to identify alcohol consumption trajectories during adulthood and relate them to risk of colorectal cancer in a large European cohort [14]. In the current study, we aimed to investigate alcohol consumption trajectories and risk of BC by using repeated measurements of alcohol intakes from 28,720 female participants in the Danish Diet, Cancer, and Health cohort. We identified and characterized alcohol consumption trajectories across adulthood using LCMM. Furthermore, we estimated the association between trajectory profiles of alcohol consumption and the risk of first primary malignant BC in postmenopausal women.

## Materials and methods

### Cohort characteristics and study population

**Fig. 1:**
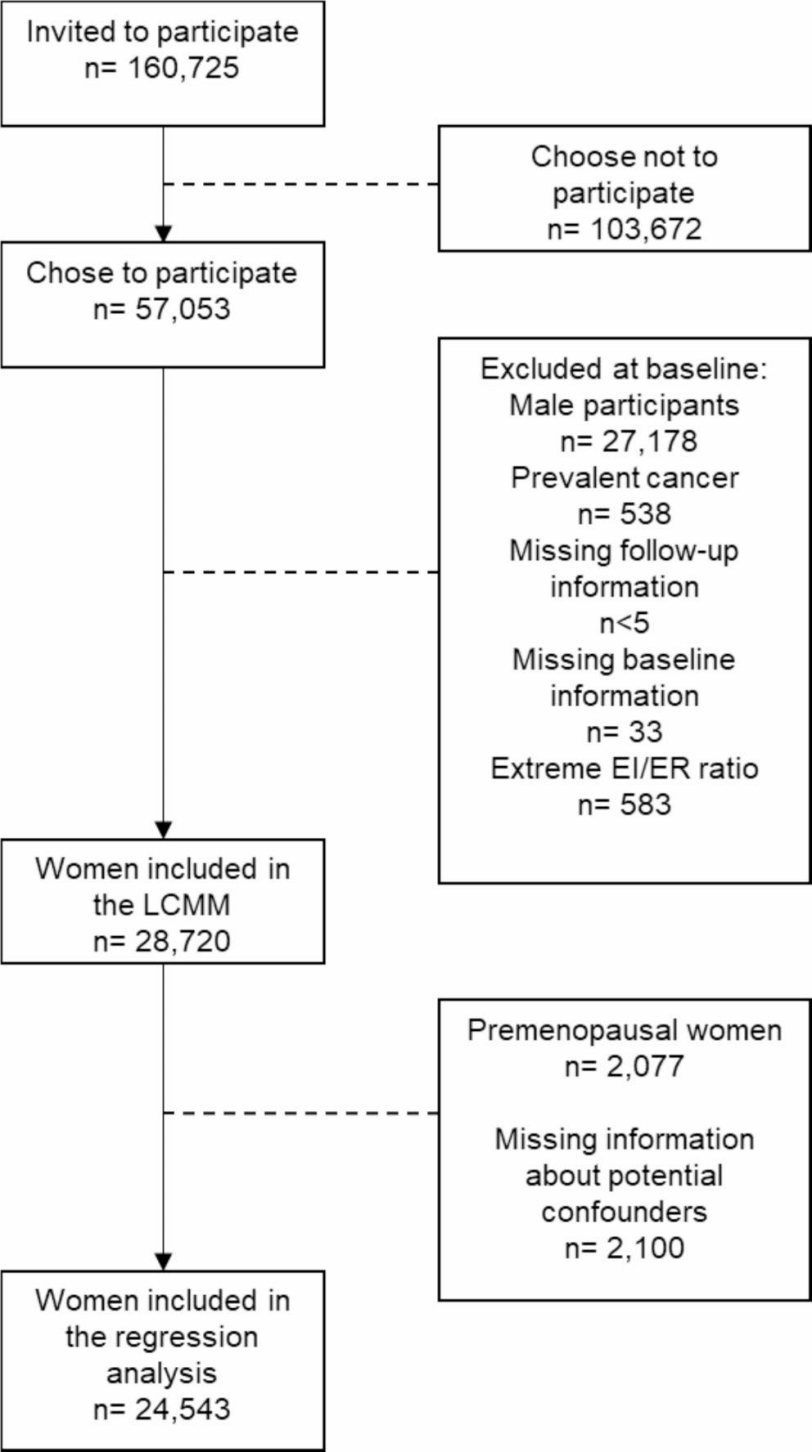
Flow of participants into the study^1^ ^1^Note: LCMM: latent class mixed model; n: number; EI/ER ratio: energy intake to energy requirement ratio

Between 1993 and 1997, 160,727 men and women were invited to participate in the Danish prospective cohort study “Diet, Cancer and Health (DCH)” in which 57,053 participants were included [15]. To be eligible for inclusion, participants had to be born in Denmark, live in the Copenhagen or Aarhus areas and with no previous cancer diagnosis registered in the Danish Cancer Registry. At enrolment, all participants filled in self-administered questionnaires about their diet and various lifestyle factors and attended a physical examination at one of two study centers located in Copenhagen and Aarhus where their anthropometrics were measured. Further details about the study can be found elsewhere [15]. For the estimation of alcohol consumption trajectories, we excluded all male participants (*n* = 27,178), women with a cancer diagnosis at baseline (*n* = 538), missing follow-up information (*n* < 5) and women without baseline information (*n* = 33) (Fig. 1). Furthermore, women with an energy ratio in the top or bottom 1% of the energy intake to calculated energy requirement distribution were excluded (*n* = 583). Thus, we used information from 28,720 women to estimate alcohol consumption trajectories. For the proportional hazard regression model, we further excluded women who were premenopausal at baseline (*n* = 2,077) and women with missing information on covariates considered potential confounding variables which include: physical activity level, smoking status, educational level, BMI, age at first full term pregnancy, age at menarche, breast feeding, and ever use hormonal replacement therapy (*n* = 2,100). In total, we included 24,543 women in the regression model (Fig. 1).

### Alcohol consumption

All participants completed a validated 192-item food frequency questionnaire (FFQ), on which they reported their average intake of food and beverages including alcohol over the last 12 months [15, 16]. Using a lifestyle questionnaire, the participants were also asked to retrospectively report their average intakes of alcohol at four periods of their lives (at the ages of 20, 30, 40, 50 years) to reflect lifetime intakes of alcohol. See Supplementary Table 1 for the number of women with available information about their alcohol intake at the different ages. The participants reported their intake in 12 categories ranging from “never” to ≥ 8 times per day. In terms of their alcohol consumption, the participants reported their average frequency of consumption of beer (3 types, in bottles of 330 ml), wine in standard glassed (125 ml), fortified wine used for drinks (glassed of 60 ml) and spirits used for drinks (30 ml) [15, 17]. The amount of alcohol (measured as ethanol) in each beverage varied according to beverage type: light beer: 8.9 g/bottle; regular beer: 12.2 g/bottle; strong beer: 17.5 g/bottle; wine: 12.5 g/glass and fortified wine: 9.3 g/drink; spirits: 9.9 g/drink [17].

### Ascertainment of menopausal status and first primary malignant female breast cancer

In this study, we focused only on incident BC among women who were postmenopausal as the number of BC cases among women pre-menopausal was considered too low to provide sufficient statistical power (*n* = 134 after exclusion of women with incomplete data on potential confounders). Menopausal status was determined at baseline and according to various information about the women’s menstrual history as described previously [18]. Women were categorised as postmenopausal if they had no periods of menses during the preceding 12 months from baseline or had undergone surgically induced menopause due to a bilateral ovariectomy. Women with no information about their menstruation history, women who reported having undergone a hysterectomy surgery or exogenous hormones use were characterised as postmenopausal if they were ≥55 years. Women were categorised as perimenopausal if they had <9 menstrual periods the preceding year or if they had had menstruation the preceding year, but not at the time of the baseline assessment. Women aged 46–55 years old were categorised as perimenopausal if information about their menstruation history was missing or they stated exogenous hormone use or having undergone a hysterectomy. In the current study, we considered perimenopausal women as being post-menopausal (*n* = 4,114 women after exclusion of women with incomplete data on potential confounders) as we assumed that these women would have reached menopause at the time of diagnosis or end of follow-up. Cases of first primary malignant BC were identified through record linkage to the Danish Cancer Registry using each participant’s unique personal identification number. First primary malignant female BC (C50) was defined according to the International Classification of Disease for Oncology.

### Covariate assessment

From the lifestyle questionnaire administrated at baseline (between 1993 and 1997), we obtained information about several lifestyle and reproductive factors which included educational level (considered a proxy for socioeconomic status), smoking status and physical activity level, age at birth of first child, age at menarche, breast-feeding history, and use of hormonal replacement therapy. The participants’ height and weight were measured by trained lab technicians. The participants’ height was measured while standing without shoes and to the nearest 0.5 cm while the participants’ weight was measured in light underwear using a digital scale to the nearest 0.1 kg. BMI was calculated as weight (kg) divided by height squared (m^2^) [15].

### Statistical analysis

To associate trajectories of alcohol consumption with the risk of BC, we adopted a multi-step analysis approach [19, 20]. First, we identified subgroups of women with distinct alcohol consumption patterns across adulthood using LCMM. Second, we used a proportional hazard model to investigate the association between the identified latent classes and risk of first primary malignant BC.

### Analysis step 1: trajectory estimation

We fitted LCMM from all available alcohol measurements (measured at baseline and at the ages 20,30,40 and 50 years) irrespectively of the participants’ menopausal status and available covariate information. All measurements of the participants’ alcohol intakes were log-transformed (log(x + 1)) to account for their non-normal distribution. We modelled the trajectories as a function of participants’ age (rescaled and recentred: (age-35/13)) assuming a quadratic time trend both at the population level and at the individual level with random effects to account for the intra-individual correlation between the alcohol measurements of the same woman. We used a quadratic time function determined based on visual inspection of 100 randomly selected women’s individual trajectories of alcohol consumption. The LCMM models were estimated within the maximum likelihood framework for a varying number of latent classes ranging from 1 to 6. To prevent convergence towards a local suboptimal maximum and to fully explore the parameter space, we ran the estimation procedure from 100 vectors of random initial parameter values based on the model parameters of the 1-class trajectory model. Furthermore, for the 5 and 6-class models, respectively, we manually specified initial values based on the model parameters obtained from models with a lower number of latent classes. We determined the most appropriate number of latent classes according to several statistical criteria: the Bayesian Information Criteria (BIC), entropy, the Integrated Classification Likelihood Criterion (ICL) and the posterior probability classification [19, 21]. Furthermore, we did not retain latent classes considered too small (<5% of the participants) as the number of BC cases within each class would be too limited for further analysis (see the supplementary method for a detailed description of the criteria). To further evaluate the goodness-of-fit of the selected model, we plotted the weighted-subject specific predicted trajectories against the weighted mean observed trajectories. After determination of the final latent class model, each woman was assigned to the trajectory class to which they had the highest posterior probability of belonging given their measures of alcohol intakes for the purpose of using standard summary statistics to describe baseline socio-demographic, lifestyle, and reproductive characteristics of the women according to the assigned latent class. We used the “GRoLTS-Checklist: Guidelines for Reporting on Latent Trajectory Studies” to guide our trajectory estimation [22].

### Analysis step 2: risk of breast cancer according to the estimated alcohol trajectory profiles

To investigate the association between the latent class structure and the risk of BC, we estimated class-specific hazard ratios (HR) and corresponding 95% confidence intervals (CI) using a parametric proportional hazard model assuming proportional hazards across latent classes. The time to diagnosis of first primary malignant BC was right censored by death, emigration, or loss to follow-up, which ever came first. We maximized the joint likelihood by keeping the parameters of the LCMM fixed while estimating the parameters of the hazard model. This allowed us to model the risk of BC according to the true latent class structure while properly accounting for the inherent classification error in the posterior assignment [19]. We fitted two parametric survival models with proportional hazards across classes. One model assuming a Weibull baseline hazard function and a second model approximating the baseline hazard by a cubic M-splines with 5 and 4 knots, respectively and compared the models according to the AIC. According to the AIC, we selected the more flexible hazard specific model with cubic M-splines with 4 knots and modelled the instantaneous risk of BC according to participant age. The estimated procedure accounted for delayed entry. We used two levels of adjustment for potential confounding factors. In Model 1, we adjusted for participant age (underlying timescale) and baseline date of recruitment into the cohort (in quintiles). In Model 2, we further adjusted for the following lifestyle and reproductive factors: physical activity level (inactive, moderately inactive, moderately active, active), smoking status (current smoker, former smoker, never smoker), educational level (primary, technical/professional school, secondary school, higher education), BMI (<18.5 kg/m^2^, 18.5–24.9 kg/m^2^, 25-<30 kg/m^2^, ≥ 30 kg/m^2^), age at first full term pregnancy (no full-term pregnancy, ≤ 21, 21–30, >30 years), age at menarche (≤12, 12–14, >14 years), breast feeding (yes, no), and ever use hormonal replacement therapy (yes, no). Potential confounding variables were selected a priori based on previous literature and a directed acyclic graph (Supplementary Fig. 2). The directed acyclic graph was created using the online resource “DAGitty” [23].

We performed all statistical analyses in R version 4.1.2 (R Foundation for Statistical Computing, Vienna, Austria), primarily using the lcmm R package, (Version 2.1.0) [24]. We used the “hlme” function for analysis step 1 and the “externVar” function for analysis step 2.

## Results

### Selection of the number of latent classes

**Fig. 2:**
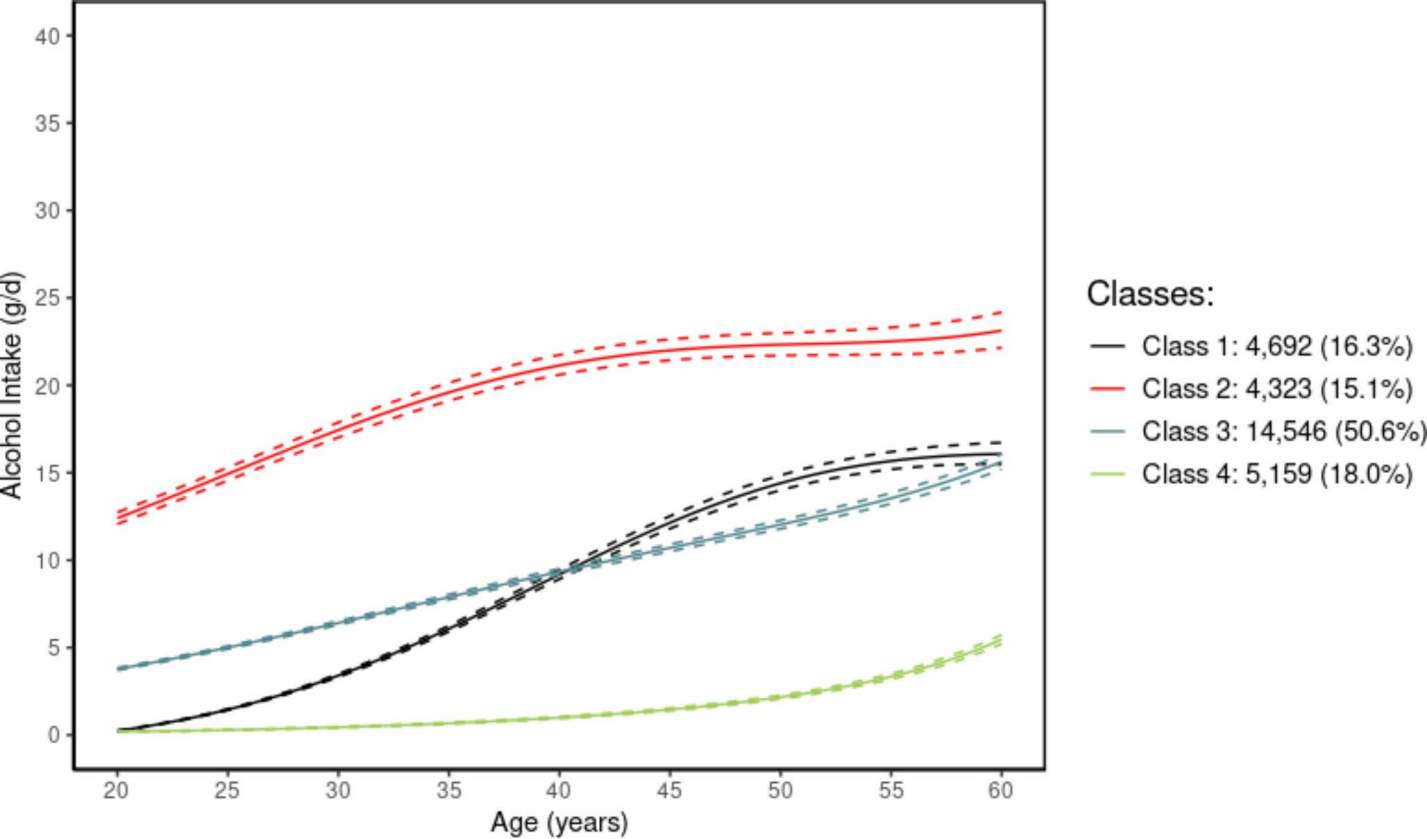
Mean predicted trajectories of alcohol consumption: 4-class model^1^ ^1^Note: Percentage numbers are rounded to the first decimal place

We saw a progressive improvement in the BIC, ICL and entropy with an increasing number of latent classes from 1 to 6 (see the supplementary result description and Supplementary Fig. 3). However, beyond 4 classes, we considered the additional classes to be too small to be meaningful for inference purposes (3.3% and 0.6%) (Supplementary Table 3). The mean posterior probability of the 4-class model was considered acceptable (≥75% in all classes) (Supplementary Table 4). Therefore, based on an overall assessment of the statistical measures, we retained the model with 4 latent classes. When we evaluated the goodness-of-fit of the 4-class model, it showed good agreement between the fitted values and the actual observations with weighted mean subject-specific predictions close to the weighted mean observations (Supplementary Fig. 5). The within-class inter-subject variability of the model fitted with random effects remained substantial with an inter-subject variance at age 35 almost twice as large as the residual variance (see parameter estimates in Supplementary Table 5) which underlines the relevance of the LCMM methodology. The predicted mean trajectories of alcohol consumption of the 4-class model are displayed in Fig. 2, while the mean predicted trajectories of alcohol consumption of models with 1–6 latent classes are shown in Supplementary Fig. 6. Visualisation of the progressive split of women into classes with increasing number of latent classes (1–6) is provided in Supplementary Fig. 7.

### Alcohol consumption trajectories

Among 28,720 women, half of the women (50.6%) had a mean trajectory of alcohol consumption characterised by an increasing low alcohol intake in early adulthood tangent to a moderate alcohol intake in mid-adulthood (Class 3), 18.0% of the women had an mean alcohol consumption trajectory characterised by continuous low intake throughout adulthood (Class 4), while 16.3% had an mean alcohol consumption trajectory characterised by a low alcohol intake throughout early-adulthood transitioning to an increasing moderate intake in mid-adulthood (Class 1). The remaining women (15.1%) had a mean alcohol consumption trajectory characterised by a continuously high alcohol intake across adulthood (Class 2) (Fig. 2). Compared to the total cohort of postmenopausal women, women who belonged to class 4 (continuously low intake of alcohol across adulthood) were more likely to have higher age at baseline and tended to have a lower average lifetime intake of alcohol, while women belonging to Class 2 (consistently high alcohol intake) tended to have a higher average lifetime alcohol intake (Table 1). Furthermore, women in Class 4 were less likely to have a higher education compared to the total cohort, while those with consistently high intakes of alcohol (Class 2) were more likely to have a higher educational level. Furthermore, women in class 4 with a consistently low alcohol intake were less likely to have used hormonal replacement therapy and to be above age 26 when they had their first full term pregnancy compared to the total cohort. However, women of Class 2 were more likely to be age >26 at their first full term pregnancy compared to the total cohort. Women in Class 4 were more likely to be never smokers, physically inactive, to be >14 years old when they had their first menstrual bleeding, and to have a BMI ≥30 kg/m^2^. Only women with a consistently high alcohol intake (Class 2) were less likely to have breast fed compared to the total cohort. Characteristics of all women and according to trajectory classes irrespectively of menopausal status and missingness on covariates are shown in Supplementary Table 6. Baseline characteristics of the total cohort of postmenopausal women and of those who were diagnosed with BC during follow-up are shown in Supplementary Table 7.

**Table 1:** Baseline socio-demographic, lifestyle, and reproductive characteristics of all postmenopausal women and according to alcohol consumption trajectories (complete cases, *n* = 24,543)

Total cohort and Alcohol consumption trajectory classes					
Characteristics^1*^	Total cohort	Class 1: low-moderate consumption with increasing intake	Class 2: Consistently high consumption	Class 3: Moderate consumption with increasing intake	Class 4: Consistently low consumption
**Number of participants**,** % (n)**	100.0 (24,543)	16.7 (4,087)	14.6 (3,577)	50.3 (12,351)	18.4 (45,298)
**Breast cancer cases**,** % (n)**	6.5 (1,591)	6.6 (269)	7.6 (272)	6.4 (787)	5.8 (263)
**Age at baseline (median**,** p10-p90)**	56.8 (51.5–63.4)	57.9 (52.0-63.7)	54.5 (51.0-61.6)	56.4 (51.4–63.1)	58.8 (52.2–64.0)
**Educational level**,** % (n)**					
Primary Technical/professional school Secondary school Higher education	32.3 (7,938) 46.3 (11,362) 11.4 (2,802) 9.9 (2,441)	36.1 (1,476) 46.6 (1,904) 9.2 (375) 8.1 (332)	19.2 (687) 46.2 (1,651) 18.3 (656) 16.3 (583)	28.7 (3,550) 48.4 (5,981) 12.2 (1,501) 10.7 (1,319)	49.1 (2,225) 40.3 (1,826) 6.0 (270) 4.6 (207)
**BMI**,** % (n)**					
<18.5 kg/m^2^ 18.5–24.9 kg/m^2^ 25-<30 kg/m^2^ ≥30 kg/m^2^	1.20 (292) 50.2 (12,311) 34.7 (8,514) 14.0 (3,426)	1.2 (48) 49.6 (2,026) 35.8 (1,462) 13.5 (551)	1.2 (43) 55.5 (1,985) 31.7 (1,134) 11.6 (415)	1.1 (137) 51.8 (6,393) 34.3 (4,233) 12.9 (1,588)	1.4 (64) 42.1 (1,907) 37.2 (1,685) 19.3 (872)
**Average lifetime intake of alcohol (g/d) (median**,** p10-p90)**^**2**^	6.6 (0.8–18.8)	6.4 (2.7–16.8)	16.9 (9.5–33.3)	7.0 (2.8–15.6)	0.5 (0.0-3.5)
**Smoking status**,** % (n)**					
Never Former Smoker	43.0 (10,562) 24.9 (6,118) 32.0 (7,863)	45.1 (1,843) 24.0 (981) 30.9 (1,263)	34,7 (1,242) 28.9 (1,033) 36.4 (1,302)	43.2 (5,338) 25.6 (3,163) 31.2 (3,850)	47.2 (2,139) 20.8 (941) 32.0 (1,448)
**Physical activity level**,** % (n)**					
Inactive Moderately inactive Moderately active Active	10.7 (2,632) 32.6 (7,994) 24.7 (6,067) 32.0 (7,850)	10.3 (421) 33.0 (1,348) 25.9 (1,058) 30.8 (1,260)	10.4 (372) 30.3 (1,085) 25.7 (920) 33.5 (1,200)	9.6 (1,191) 33.3 (4,107) 25.1 (3,105) 32.0 (3,948)	14.3 (648) 32.1 (1,454) 21,7 (984) 31.8 (1,442)
**Age at menarche**,** % (n)**					
≤12y 13-14y >14y	23.4 (5,741) 49.5 (12,138) 27.2 (6,664)	23.9 (975) 48.0 (1,960) 28.2 (1,152)	23.9 (855) 51.7 (1,848) 24.4 (874)	23.3 (2,876) 49.8 (6,153) 26.9 (3,322)	22.9 (1,035) 48.1 (2,177) 29.1 (1,316)
**Age at first full-term pregnancy**,** n (%)**					
No full-term pregnancy ≤21y 22-26y >26y	11,9 (2,917) 29.6 (7,268) 38,9 (9,546) 19,6 (4,812)	9.7 (397) 34.2 (1,398) 40.2 (1,643) 15.9 (649)	17.2 (614) 22.3 (798) 35.8 (1,279) 24.8 (886)	11.8 (1,462) 27.1 (3,345) 40.2 (4,967) 20.9 (2,577)	9.8 (444) 38.1 (1,727) 36.6 (1,657) 15.5 (700)
**Ever use of hormonal replacement therapy**,** n (%)**					
Yes No	47.9 (11,747) 52.1 (12,796)	49.2 (2,012) 50.8 (2,075)	51.0 (1,826) 49.0 (1,751)	48,1 (5,946) 51.9 (6,405)	43.4 (1,963) 56.6 (2,565)
**Ever breastfed**,** n (%)**					
Yes No	82.0 (20,132) 18.0 (4,411)	84.3 (3,444) 15.7 (643)	76.5 (2,735) 23.5 (842)	82.5 (10,193) 17.5 (2,158)	83.0 (3,760) 17.0 (768)

Note: N/n: Numbers; p: Percentile; %: Percentage; BMI: Body Mass Index; y: Years; g/d: gram per day. ^1^ Complete case data, ^2^Information only available for 4,087, 3,576, 12,290, and 4,503 women, respectively; *Numbers are rounded to the first decimal place

### Associations between trajectories of alcohol consumption and risk of first primary malignant breast cancer

During a median follow-up time of 16.5 years, a total of 1,591 BC cases were diagnosed. In the multivariable model, when using women with an alcohol consumption trajectory characterised by a consistently low alcohol intake (mean profile < 6 g/day, class 4) as the reference group, women with a a continuously high (mean profile > 10 g/day, class 2) alcohol intake across adulthood had a higher risk of BC (HR: 1.65; 95%CI 1.35–2.03) (Table 2). In contrast, we found no association between the two mean trajectories of alcohol consumption characterized by a lower alcohol intake in early adulthood followed by an increased intake of alcohol throughout adulthood compared to the reference (HR: 1.18, 95%CI: 0.94–1.48, HR: 1.15, 95%Cl: 0.94–1.40, respectively). Compared to Model 1, the point estimates obtained from the fully adjusted model were attenuated.

**Table 2:** Associations between alcohol consumption trajectories and risk of breast cancer among postmenopausal women

Outcome	Breast cancer cases	Model 1		Model 2	
Breast cancer	Women/cases (*N*/*n*)	HR	95%CI	HR	95% CI
Class 1: Low to moderate consumption with increasing intake	4,087/269	1.24	1.01–1.52	1.18	0.94–1.48
Class 2: Consistently high consumption	3,577/272	1.85	1.53–2.23	1.65	1.35–2.03
Class 3: Moderate consumption with increasing intake	12,351/787	1.21	1.03–1.42	1.15	0.94–1.40
Class 4: Consistently low consumption.	4,528/263	1.00	Ref.	1.00	Ref.

Note: N/n: total number of participants in the class/number of cases in the class; HR: Hazard ratio; 95%CI: 95% confidence interval; ref: reference

The hazard ratios were estimated in a proportional hazard model that accounted for the uncertainty in the posterior class assignment

Model 1: adjusted for participant age (underlying time scale) and date of recruitment (in quintiles)

Model 2: further adjusted for physical activity level (inactive, moderately inactive, moderately active, active), smoking status (never, former, smoker), educational level (primary, technical/professional school, secondary school, higher education), BMI (<18.5 kg/m^2^, 18.5–24.9 kg/m^2^_,_ 25-<30 kg/m^2^, ≥30 kg/m^2^), age at menarche (≤12y, 13-14y, >14y), breast fed (yes,no), age at first full term pregnancy (no full-term pregnancy, ≤21y, 22-26y, >26y), and ever use of hormonal replacement therapy (yes, no)

## Discussion

Based on up to 5 repeated measurements of the participants’ alcohol intake with an average of 4.98 measurements per woman assessed across adulthood among 28,720 women, we identified 4 different mean profiles of alcohol consumption trajectories. Women characterized by a mean alcohol profile of >10 g/day had greater risk of first primary malignant BC compared to women with a mean alcohol profile of <6 g/day during adulthood. We found no evidence of associations between the remaining alcohol consumption trajectories and risk of BC when compared to women with a mean alcohol consumption trajectory characterized by a consistently low intake of alcohol.

### Strengths and limitations

This study had a large study population with a large number of cases, a long follow-up time, minimal loss to follow-up and detailed and repeated information on women’s alcohol intakes across adulthood. Therefore, we were able to investigate associations between exposure to alcohol and risk of BC while simultaneously capturing the variation in drinking behavior across adulthood. In contrast to the few existing studies on associations between alcohol consumption trajectories and risk of BC, we fitted a LCMM instead of a GBTM, which is a commonly used approach in epidemiologic studies conducting trajectory analysis [25–29]. Although GBTM and LCMM rely on the same core assumptions, the LCMM captures within-class variation by introducing subject-specific random effects to the model [12]. Heterogeneity across women with similar trajectory profiles is not accounted for in the GBTM approach as that model assumes that individual trajectories remain homogeneous within each latent class. The validity of such an assumption may be questionable in many applications with several exposure measurements of the same study participant [13]. However, we acknowledge that the LCMM is a more complicated model that can result in convergence issues, including computational constraints in very large sets of data. Thus, both modelling approaches can provide important insights interpreted in the light of their underlying assumptions [22]. Furthermore, irrespective of the latent class trajectory modelling approach employed, the posterior assignment of participants is subject to classification error and does not reflect the true underlying latent class structure, leading to incorrect inference if used directly as exposure variable in a regression model. This is especially an issue when the discriminatory power of the latent class model is limited [20, 30]. In this study, we accounted for the uncertainty related to the class membership assignment in the proportional hazard models by adopting a modelling approach that accounted for the classification error in the exposure [19]. In this study, the ascertainment of cancer cases during follow-up was conducted through record linkage to the Danish Cancer Registry, which minimize the risk of selection bias due to loss to follow-up. Moreover, only 162 women did not report their alcohol intake at the ages of 20, 30, 40 and 50 years and the measurements of these women’s alcohol intake at the specific timepoints were not included in the LCMM estimation (Supplementary Table 1). Hence, the small number of missing observations are unlikely to have resulted in substantial bias. This study is also prone to limitations. Data on the women’s alcohol intake was self-reported, and retrospectively collected, therefore, likely subject to measurement errors. In the context of alcohol consumption, this may have resulted in under-reporting of alcohol intake during adulthood especially among heavy consumers [31]. Therefore, we cannot rule out systematic errors of the exposure. However, due to the prospective nature of the study, measurement errors would be non-differential with respect to BC occurrence. Furthermore, random measurement errors were accounted for in the LCMM under the assumption that they were homoscedastic and non-differential. While the baseline the FFQ was validated [16], no gold standard to assess the validity of alcohol intake measurements during early to mid-adulthood is available. In the study, women who abstained from alcohol were included in trajectory estimation. These women were probably assigned to the latent class of women with a consistently low intake of alcohol, which was used as the reference group in the proportional hazard model. An unknown proportion of these women may abstain from alcohol due to health-related issues associated with their risk of BC. Grouping these women with women with a consistently low intake of alcohol may raise the baseline rate of BC in the reference group, thus leading to diminished contrast to the other classes. Furthermore, as menopausal status was determined at baseline at not at BC diagnosis, which is more relevant in an etiological context, we considered perimenopausal women as being post-menopausal based on the assumption that these women would have entered menopause at time of diagnosis or at the end of follow-up. However, we cannot rule out that some of these women were not post-menopausal resulting in misclassification of menopausal status. Finally, although we adjusted the analyses for several potential confounding variables, we cannot rule out that residual confounding may have impacted the results.

### Comparison with other studies

In 2018, the World Cancer Research Fund (WCRF) reported a higher risk of BC of 9% per 10 g/day intake of alcohol among post-menopausal women in their dose-response meta-analysis of 22 studies (RR: 1.09, 95%CI: 1.07,1.12) [5]. Despite this large body of evidence, only few studies have investigated associations between alcohol consumption trajectories and risk of BC. In a Spanish study of 1,017 case-control pairs, using a GBTM approach, the authors identified 4 distinct alcohol consumption profiles [9]. Similar to the trajectories observed here, the authors identified one trajectory of women with a trajectory profile of consistently low alcohol intake but with a slightly lower mean intake (<5 g/day vs. <6 g(day). Furthermore, the authors identified a second trajectory profile of women characterized by a low alcohol intake in adolescence (<5 g/day) with a moderate intake throughout adulthood (5-<15 g/day), which is similar to our classes 1 and 3. Compared to consistently low alcohol intakes, no associations with BC were found (OR: 1.22, 95%CI: 0.83,1.79) [9], which are similar to our results for classes 1 and 3 [9]. Similarly, a prospective cohort study of 22,767 pre- and post-menopausal women in which 3 alcohol trajectories were identified using a GBTM found no higher risk of BC (HR: 0.95, 95%CI: 0.84,1.06) when comparing a trajectory characterized by a stable low intake of alcohol through adulthood (approx. 5–10 g/day) to lifetime abstainers (<2.2 g/day) [8]. In the Spanish case-control study a moderate alcohol intake (5-<15 g/day) from adolescence to youth that decreased throughout adulthood was associated with higher odds of BC (OR: 1.79, (95%Cl: 1.02, 3.15) among postmenopausal women compared to consistently low intake. This could indicate that a relatively high alcohol intake early in life followed by a lower alcohol intake throughout adulthood bears an impact on BC risk [9]. Associations between alcohol consumption in youth and early adulthood and risk of BC have been reported previously although results are inconclusive [32]. On the other hand, all three trajectory studies to date, including our own, have observed that consistently high alcohol consumption patterns across adulthood were associated with risk of BC, whether compared to either lifetime abstainers or consistently low intakes in adulthood [8, 9]. This indicates that a mean alcohol consumption pattern of more than 5 g/day from adolescence and across adulthood may play a profound role in relation to risk of BC irrespective of fluctuations in alcohol consumption during the remaining part of life.

## Conclusion

Adopting a latent class mixed modelling approach to estimate alcohol consumption trajectories, we identified four distinct patterns of alcohol consumption behavior. The results of this study suggest that post-menopausal women who have an alcohol consumption trajectory characterized by a continuously high consumption of alcohol throughout adulthood have a higher risk of developing first primary malignant BC.

## Electronic supplementary material

Below is the link to the electronic supplementary material.

Supplementary Material 1
