# Supplementary material for: Alcohol consumption trajectories and risk of breast cancer among postmenopausal women: a Danish cohort study
Source: Eur J Epidemiol. 2024 Dec 4;39(12):1353–62. doi: 10.1007/s10654-024-01179-5 (PMC11680656; doi:10.1007/s10654-024-01179-5)
Supplement: Supplementary file 1 — Supplementary Material 1 [file 10654_2024_1179_MOESM1_ESM.docx]

**Supplementary Information: Alcohol consumption trajectories and risk of breast cancer among postmenopausal women: A Danish cohort study**

Christian S. Antoniussen^1^, Cécile Proust-Lima^2^, Daniel B. Ibsen^1,3,4^, Anja Olsen^1,5^, Kim Overvad^1^, Anne Tjønneland^5^, Pietro Ferrari^6*^, Christina C. Dahm^1*^

^1^Department of Public Health, Aarhus University, Aarhus, Denmark; ^2^University of Bordeaux, Inserm, Bordeaux Population Health Research Center, Bordeaux, France; ^3^Steno Diabetes Center Aarhus, Aarhus University Hospital, Aarhus, Denmark; ^4^Department of Nutrition, Exercise and Sports, University of Copenhagen, Copenhagen, Denmark; ^5^Danish Cancer Institute, Copenhagen, Denmark; ^6^Nutrition and Metabolism Branch, International Agency for Research on Cancer (IARC), World Health Organization, Lyon, France.

**The authors contributed equally*

**Corresponding author:** Christina C. Dahm, Department of Public Health, Aarhus University, Aarhus, Denmark. E-mail: [ccd@ph.au.dk](mailto:ccd@ph.au.dk), ORCID: <https://orcid.org/0000-0003-0481-2893>

**Journal:** European Journal of Epidemiology

Supplementary Information

[Supplementary Table 1: Number of participants with information about their alcohol intake and median alcohol intake at baseline and at the ages of 20, 30, 40, 50 years, respectively 3](#_Toc177545181)

[Supplementary Method Description: Statistical criteria used for model selection and model performance assessment 4](#_Toc177545182)

[Supplementary Fig 2: Association between longitudinal alcohol consumption trajectories and risk of first primary malignant breast cancer among post-menopausal women: a directed acyclic graph^1^ 4](#_Toc177545183)

[Supplementary Result Description 6](#_Toc177545184)

[Supplementary Fig. 3: Comparison of models with 1 to 6 classes in terms of the Bayesian Information Criterion, Entropy, and the Integrated Classification likelihood Criterion 6](#_Toc177545185)

[Supplementary Table 3: Mean posterior class-membership probability (%) in each class of the 4-class model 7](#_Toc177545186)

[Supplementary Table 4: Model performance assessment and goodness-of-fit measures of models with 1-6 latent classes 7](#_Toc177545187)

[Supplementary Fig. 5: Plot of the mean subject-specific predictions versus the mean observations of the 4-class model weighted by the posterior individual probability of belonging to each latent class 8](#_Toc177545188)

[Supplementary Table 5: Model parameters of the 4-class model 9](#_Toc177545189)

[Supplementary Fig. 6: Mean predicted alcohol consumption trajectories: latent classes ranging from 1-6^1^ 10](#_Toc177545190)

[Supplementary Fig. 7: Sankey plot showing the progressive split of women into latent classes: 1-6 classes 11](#_Toc177545191)

[Supplementary Table 6: Baseline socio-demographic, lifestyle, and reproductive characteristics of the total cohort and according to trajectory classes irrespectively of menopausal status and missingness on covariates, n=28,720 12](#_Toc177545192)

[Supplementary Table 7: Baseline socio-demographic, lifestyle, and reproductive characteristics of postmenopausal women in the Diet, Cancer, and Health cohort and of those who were diagnosed with first primary malignant breast cancer during follow-up, separately (complete cases) 14](#_Toc177545193)

[References 15](#_Toc177545194)

# Supplementary Table 1: Number of participants with information about their alcohol intake and median alcohol intake at baseline and at the ages of 20, 30, 40, 50 years, respectively

| **Time point** | **n/N** | **Alcohol intake (g/d)^*^** |
| --- | --- | --- |
| **At baseline** | 28,720/28,720 | 9.4 (1.1-34.5) |
| **Age 20 y** | 28,558/28,720 | 2.3 (0.0-9.7) |
| **Age 30 y** | 28,558/28,720 | 4.5 (0.0-15.2) |
| **Age 40 y** | 28,558/28,720 | 6.8 (0.0-21.7) |
| **Age 50 y** | 28,558/28,720 | 8.0 (0.0-26.3) |
| Note: *Median (10th-90th percentile); y: years; n/N: participants with available information/ total number of participants included in the latent class mixed model; g/d: gram per day | | |

# Supplementary Method Description: Statistical criteria used for model selection and model performance assessment

For the model assessment, we used several statistical criteria including the Bayesian Information Criterion, the Entropy of the model Integrated Classification likelihood criterion (ICL), and the mean posterior probability. When evaluating the model fit, lower BIC values were considered indicative of a better fit of the model to the data. We evaluated the discriminatory performance of the model using an entropy assessment. An entropy as close to 0.80 as possible or above was considered acceptable, indicating that the classes were well differentiated [1, 2]. To assess the balance between model fit, model complexity and the discriminatory power of the models we calculated the ICL. A lower ICL indicated a balance between goodness-of-fit and discriminatory power [1]. We calculated the mean posterior probability classification to get a measure of the most likely class assignment. A mean posterior probability corresponding to ≥70% in all classes was regarded acceptable [2].

# Supplementary Fig 2: Association between longitudinal alcohol consumption trajectories and risk of first primary malignant breast cancer among post-menopausal women: a directed acyclic graph^1^

**Unadjusted model**

The red circles represent potential confounding variables, while the light green circle and blue circle represent the exposure and outcome variable of interest, respectively. The Minimal sufficient adjustment set includes: age (used as underlying timescale in the time-to-event analysis), age at first full-term pregnancy, age at menarche, BMI, breast feeding, hormonal replacement therapy, physical activity level, smoking status, and educational level. The variable “Alcohol consumption” covers alcohol intake across early and mid-adulthood.


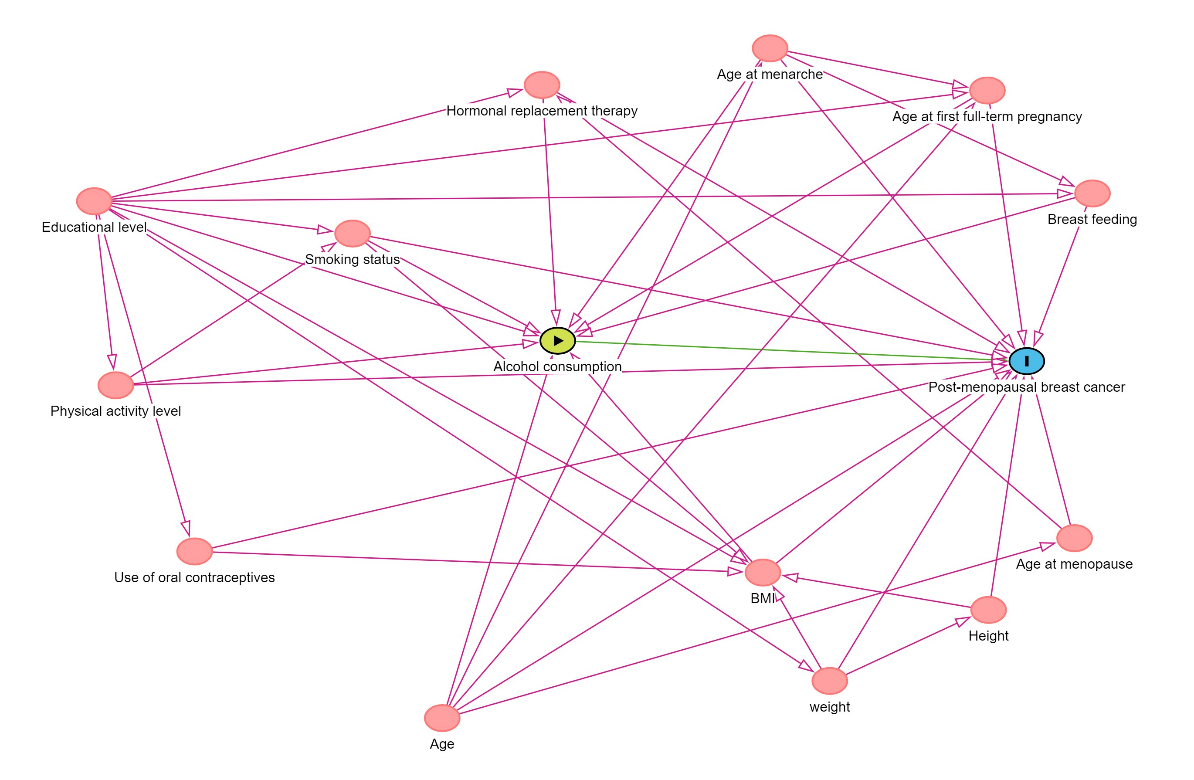


**Fully adjusted model:**

**
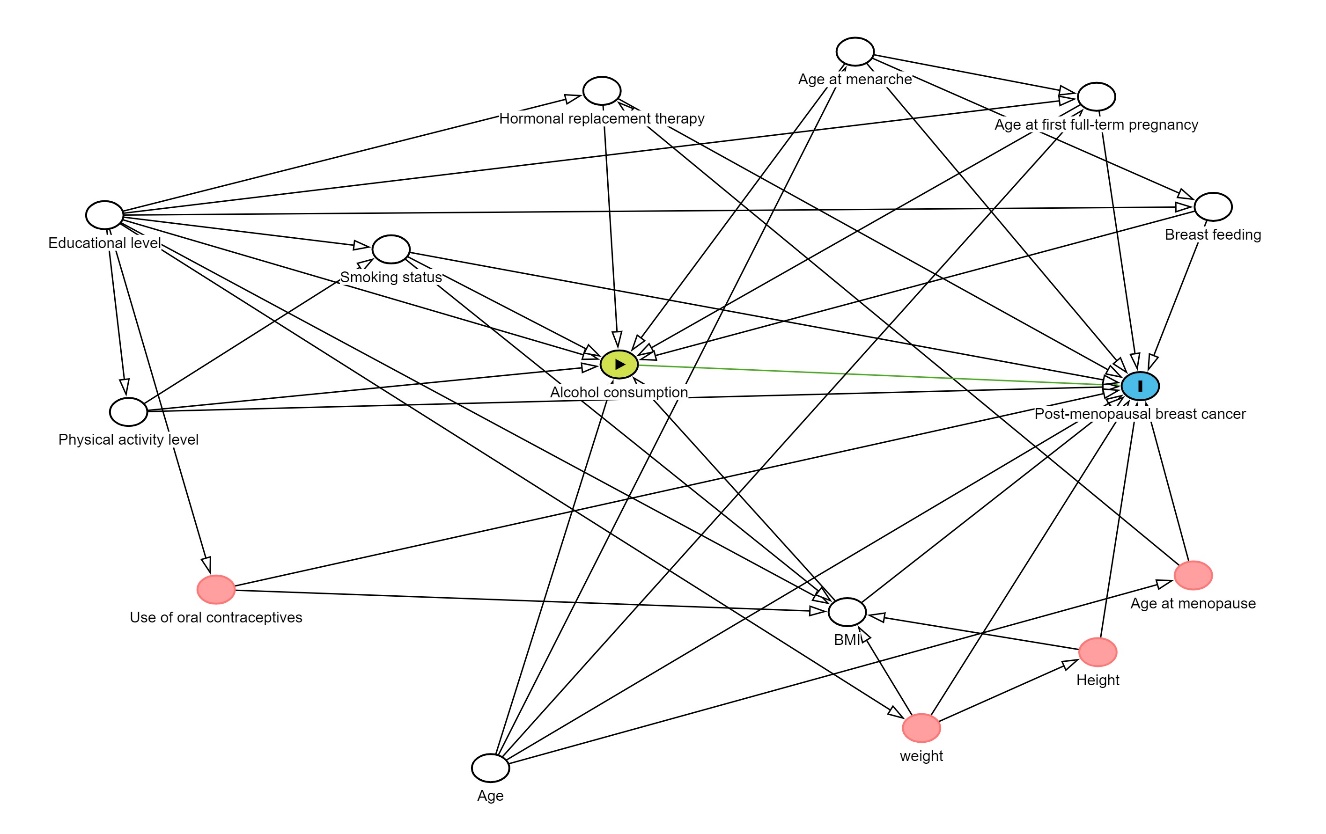
**The white circles represent adjustment variables while the red circles represent potential confounding variables. The light green circle and blue circle represent the exposure and outcome variable of interest, respectively. The variable “Alcohol consumption” covers alcohol intake across early and mid-adulthood.

^1^The figure was created created using the online resource “DAGitty” [3].

# Supplementary Result Description

As seen by the continuous decrease in BIC, the goodness-of-fit was improved when the number of latent classes were increased. Although the entropy was higher for the 5 and 6 class model compared to the model with 4 classes, we considered the additional classes to be too small (<5%). Similar tendency can be seen for the ICL. Although, the ICL favoured the 6-class model, we considered the additional classes to be too small to be meaningful for inference purposes. The gradually increase in entropy from the 4-class model to the 5 and 6 latent class model and the corresponding decrease in ICL from the 4-class model to the 6-class model is probably due to identification of small but distinct trajectories of alcohol consumption which result in an increase in the discriminative power of the model. Furthermore, the mean posterior probability of the model with 4 classes was >70% in all classes as shown in Supplementary Table 3. An overview of the loglikelihood of each model, the different criteria to assess the model performance and the percentages distribution of women into classes of each model appears from Supplementary Table 4.

# Supplementary Fig. 3: Comparison of models with 1 to 6 classes in terms of the Bayesian Information Criterion, Entropy, and the Integrated Classification likelihood Criterion


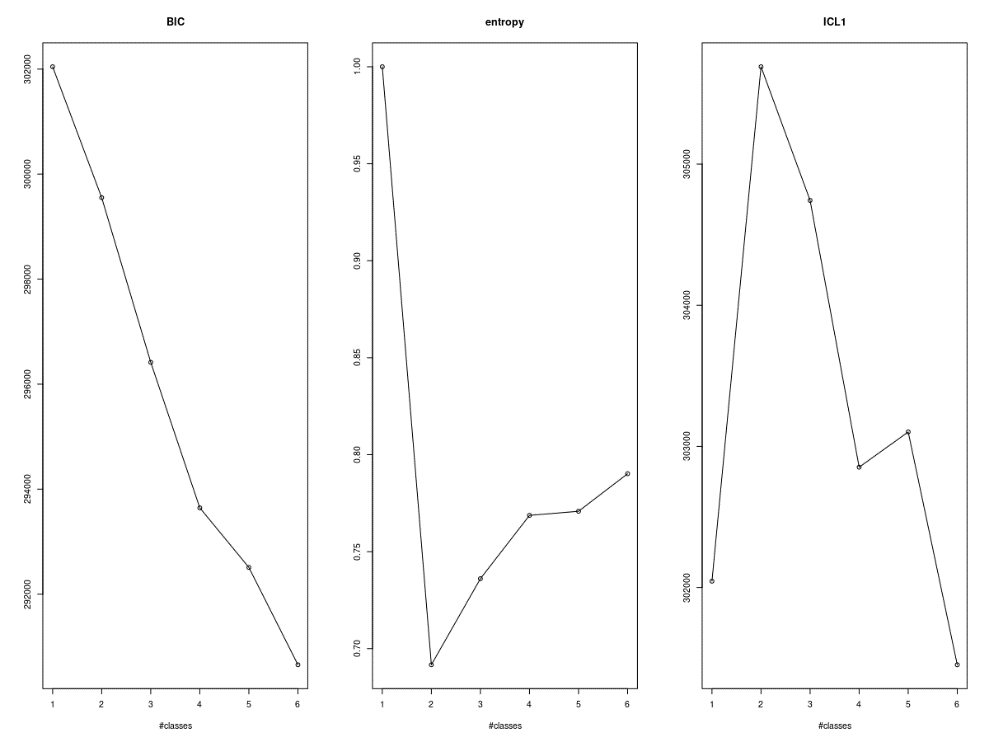


# Supplementary Table 3: Mean posterior class-membership probability (%) in each class of the 4-class model

| **Classes*** | **Class 1** | **Class 2** | **Class 3** | **Class 4** |
| --- | --- | --- | --- | --- |
| **Class 1** | 86.4 | 0.0 | 3.0 | 10.6 |
| **Class 2** | 0.0 | 84.3 | 15.7 | 0.0 |
| **Class 3** | 3.8 | 7.2 | 87.0 | 2.0 |
| **Class 4** | 8.2 | 0.0 | 2.7 | 89.0 |
| Note:; *Numbers are rounded to the first decimal place. | | | | |

# Supplementary Table 4: Model performance assessment and goodness-of-fit measures of models with 1-6 latent classes

| **Model*** | **Loglik** | **BIC** | **Entropy** | **ICL1** | **Class 1 (%)** | **Class 2 (%)** | **Class 3 (%)** | **Class 4 (%)** | **Class 5 (%)** | **Class 6 (%)** |
| --- | --- | --- | --- | --- | --- | --- | --- | --- | --- | --- |
| **Model 1 (1 class)** | -150971.1 | 302044.8 | 1.00000 | 302044.8 | 100.0 |  |  |  |  |  |
| **Model 2 (2 classes** | -149704.0 | 299551.7 | 0.69168 | 305689.4 | 56.2 | 43.8 |  |  |  |  |
| **Model 3 (3 classes)** | -148115.1 | 296415.0 | 0.73608 | 304742.3 | 60.4 | 19.2 | 20.4 |  |  |  |
| **Model 4 (4 classes)** | -146708.9 | 293643.6 | 0.76871 | 302852.4 | 16.3 | 15.1 | 50.6 | 18.0 |  |  |
| **Model 5 (5 classes)** | -146120.9 | 292508.7 | 0.77082 | 303101.9 | 15.2 | 24.5 | 3.1 | 39.5 | 17.7 |  |
| **Model 6 (6 classes)** | -145176.3 | 290655.2 | 0.79018 | 301452.5 | 15.1 | 24.6 | 3.3 | 0.6 | 38.7 | 17.8 |
| Note: BIC: Bayesian information criterion; Loglik: Log-likelihood; ICL1: Integrated Classification Likelihood Criterion; %: percentages; *Numbers are rounded to the first decimal place except for the entropy measure. | | | | | | | | | | |

#
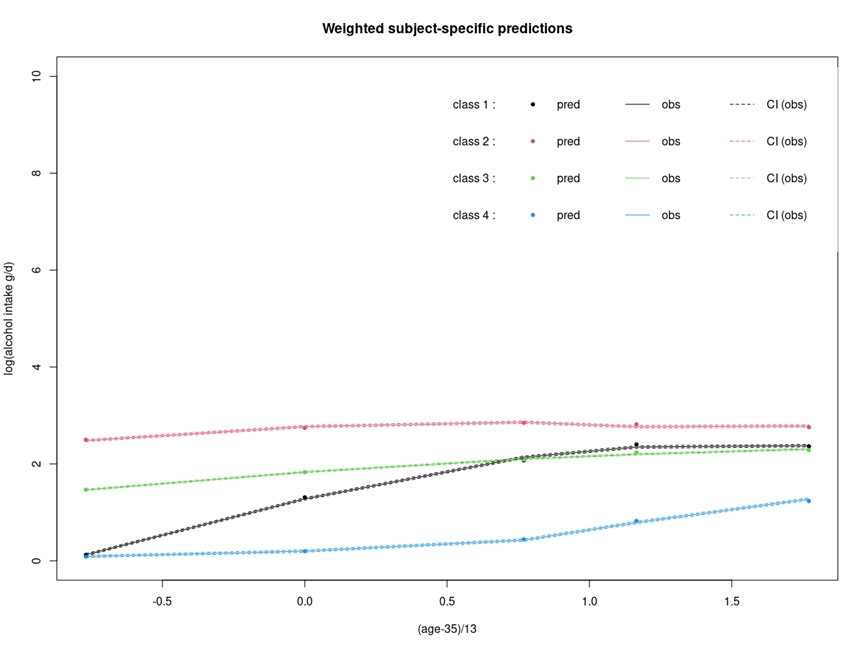
Supplementary Fig. 5: Plot of the mean subject-specific predictions versus the mean observations of the 4-class model weighted by the posterior individual probability of belonging to each latent class

# Supplementary Table 5: Model parameters of the 4-class model

| **Fixed effect in the class-membership model*** | | | | |
| --- | --- | --- | --- | --- |
|  | **Coefficient** | **Standard error** | **Wald** | **P-value** |
| Intercept class 1 | -0.06689 | 0.03370 | -1.985 | 0.04716 |
| Intercept class 2 | -0.13508 | 0.03426 | -3.943 | 0.00008 |
| Intercept class 3 | 0.92930 | 0.02216 | 41.932 | <0.0001 |
| **Fixed effect in the longitudinal model** | | | | |
|  | **Coefficient** | **Standard error** | **Wald** | **P-value** |
| Intercept class 1 | 1.74363 | 0.01262 | 138.150 | 0.00000 |
| Intercept class 2 | 2.81097 | 0.01260 | 223.018 | 0.00000 |
| Intercept class 3 | 1.97081 | 0.00747 | 263.907 | 0.00000 |
| Intercept class 4 | 0.30258 | 0.01271 | 23.800 | 0.00000 |
| Age class 1 | 0.98828 | 0.00688 | 143.565 | 0.00000 |
| Age class 2 | 0.13755 | 0.00763 | 18.039 | 0.00000 |
| Age class 3 | 0.33248 | 0.00370 | 89.967 | 0.00000 |
| Age class 4 | 0.31856 | 0.00757 | 42.106 | 0.00000 |
| I(age^2^) class 1 | -0.36277 | 0.00546 | -66.433 | 0.00000 |
| I(age^2^) class 2 | -0.11526 | 0.00504 | -22.849 | 0.00000 |
| I(age^2^) class 3 | -0.09002 | 0.00259 | -34.798 | 0.00000 |
| I(age^2^) class 4 | 0.11358 | 0.00465 | 24.415 | 0.00000 |
| **Variance-covariance matrix of the random-effects** | | | | |
|  | **Intercept** | **Age** | **I(age^2^)** | - |
| Intercept | 0.27000 | - | - | - |
| Age | 0.09960 | 0.08854 | - | - |
| I(age^2^) | -0.06055 | 0.00226 | 0.02525 | - |
| **Residual standard error** | | | | |
| Coefficient | 0.39821 | - | - | - |
| ^*^Class 4 is the reference class | | | | |

# Supplementary Fig. 6: Mean predicted alcohol consumption trajectories: latent classes ranging from 1-6^1^


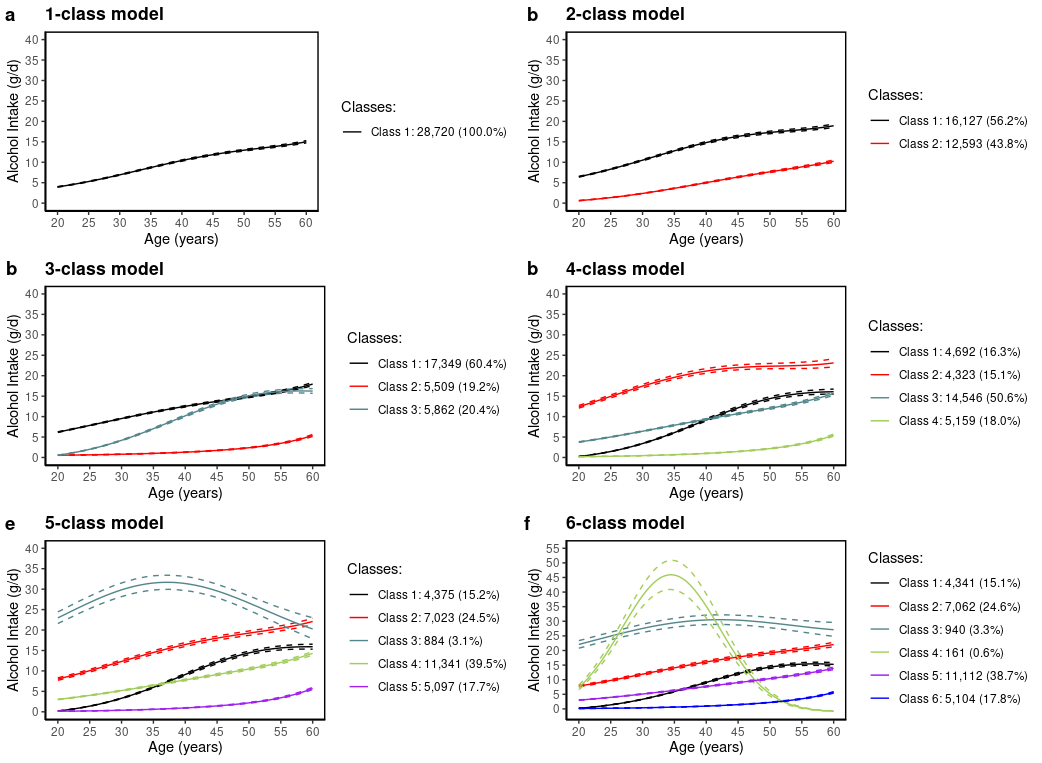


^1^Note: Percentage numbers are rounded to the first decimal place.

# Supplementary Fig. 7: Sankey plot showing the progressive split of women into latent classes: 1-6 classes


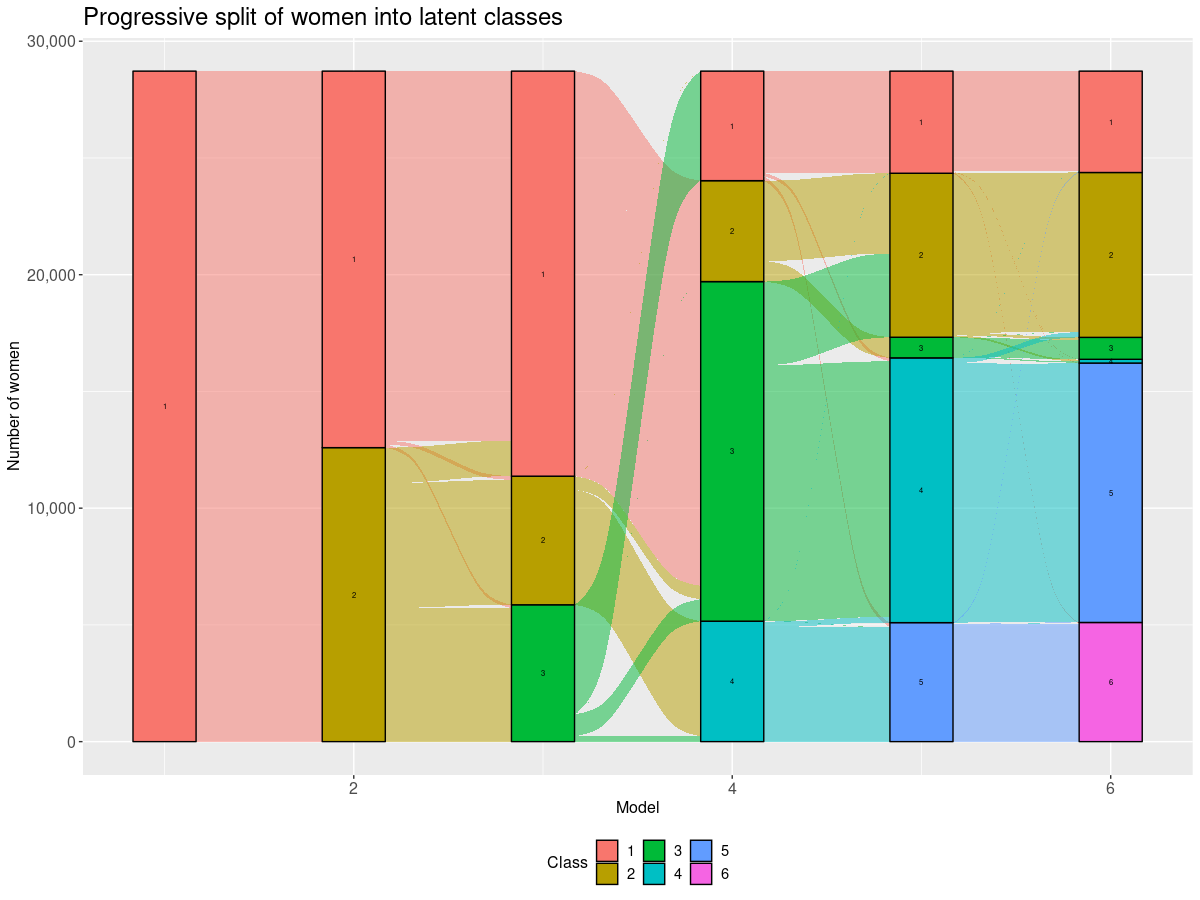


# Supplementary Table 6: Baseline socio-demographic, lifestyle, and reproductive characteristics of the total cohort and according to trajectory classes irrespectively of menopausal status and missingness on covariates, n=28,720

| **Total cohort and Alcohol consumption trajectory classes** | | | | | |
| --- | --- | --- | --- | --- | --- |
| **Characteristics*** | **Total cohort** | **Class 1: Low-moderate consumption with increasing intake** | **Class 2: Consistently high consumption** | **Class 3: Moderate consumption with increasing intake** | **Class 4: Consistently low consumption** |
| **Number of participants, % (n)** | 100,0 (28,720) | 16.3 (4,692) | 15.1 (4,323) | 50.6 (14,546) | 18.0 (5,159) |
| **Breast cancer cases, % (n)** | 6.5 (1,869) | 6.6 (312) | 7.5 (325) | 6.4 (933) | 5.8 (299) |
| **Age at baseline (median, p10-p90)**  *Missing (n)* | 56.3 (51.2-63.2)  0 | 57.5 (51.6-63.6)  0 | 54.1 (50.8-61.4)  0 | 55.9 (51.2-63.0)  0 | 58.5 (51.8-64.0)  0 |
| **Menopausal status, % (n)**  Premenopausal  Postmenopausal  *Missing (n)* | 7.2 (2,077)  92.8 (26,643)  0 | 5.2 (242)  94.8 (4,450)  0 | 10.4 (449)  89.6 (3,874)  0 | 8.1 (1,176)  91.9 (13,370)  0 | 4.1 (210)  95.9 (4,949)  0 |
| **Educational level, % (n)**  Primary  Technical/ professional school  Secondary  Higher education  *Missing (n)* | 31.3 (8,975)  46.6 (13,371)  11.8 (3,395)  10.2 (2,937)  <50 | 35.6 (1,665)  46.9 (2,195)  9.4 (438)  8.2 (384)  10 | 18.1 (781)  46.0 (1,985)  18.8 (814)  17.1 (739)  <5^1^ | 27.9 (4,055)  48.8 (7,085)  12.5 (1,816)  10.8 (1,566)  24 | 48,0 (2,474)  40.9 (2,106)  6.3 (327)  4.8 (248)  <5^1^ |
| **BMI, % (n)**  <18.5 kg/m^2^  18.5-24.9 kg/m^2^  25-<30 kg/m^2^  ≥30 kg/m^2^  *Missing (n)* | 1.2 (346)  50.5 (14,508)  34.4 (9,889)  13.8 (3,977)  0 | 1.3 (61.0)  49.7 (2,331)  35.5 (1,668)  13.5 (632)  0 | 1.1 (49)  55.3 (2,389)  32.1 (1,386)  11,5 (499)  0 | 1.1 (163)  52.3 (7,609)  33.8 (4,918)  12.8 (1,856)  0 | 1.4 (73)  42.2 (2,179)  37.2 (1,917)  19.2 (990)  0 |
| **Average lifetime intake of alcohol (g/d) (median, p10-p90)**  *Missing (n)* | 6.7 (0.8-19.0)  162 | 6.4 (2.7-10.6)  0 | 16.8 (9.5-33.0)  <5^1^ | 7.0 (2.8-15.5)  118 | 0.5 (0.0-3.5)  43 |
| **Smoking status, % (n)**  Never  Former  Smoker  *Missing (n)* | 43.8 (12,563)  24.7 (7,074)  31.5 (9,021)  62 | 45.7 (2,136)  23.7 (1,110)  30.6 (1,433)  13 | 36.1 (1,554)  28.9 (1,245)  35.1 (1,511)  13 | 44.2 (6,416)  25.3 (3,678)  30.5 (4,431)  21 | 47.8 (2,457)  20.2 (1,041)  32.0 (1,646)  15 |
| **Physical activity level, % (n)**  Inactive  Moderately inactive  Moderately active  Active  *Missing (n)* | 10.6 (3,050)  32.2 (9,235)  24.9 (7,148)  32.3 (9,265)  <30 | 10.5 (491)  32.6 (1,529)  25.7 (1,204)  31.3 (1,467)  <5* | 10.0 (431)  30.6 (1,320)  25.9 (1,117)  33.6 (1,452)  <5^1^ | 9.6 (1,392)  32.7 (4,749)  25.4 (3,695)  32.3 (4,694)  16 | 14.3 (736)  31.7 (1,637)  22.0 (1,132)  32.0 (1,652)  <5^1^ |
| **Age at menarche, % (n)**  ≤12y  13-14y  >14y  *Missing (n)* | 23.4 (6,468)  49.6 (13,723)  27.1 (7,503)  1,026 | 23.7 (1,073)  48.3 (2,187)  28.0 (1,266)  166 | 24.1 (1,003)  51.5 (2,144)  24.5 (1,019)  157 | 23.2 (3,254)  49.9 (7,008)  26.9 (3,782)  502 | 23.0 (1,138)  48.1 (2,384)  29.0 (1,436)  201 |
| **Age at first full-term pregnancy, n (%)**  No full-term pregnancy  ≤21y  22-26y  >26y  *Missing (n)* | 11.8 (3,331)  29.3 (8,289)  39.0 (11,049)  20.0 (5,669)  382 | 9.7 (450)  34.3 (1,587)  40.2 (1,864)  15.8 (73 1)  60 | 16.9 (724)  21.7 (928)  35.7 (1,527)  25.7 (1,099)  45 | 11.6 (1,666)  26.9 (3,856)  40.4 (5,801)  21.1 (3,030)  193 | 9.7 (491)  37.8 (1,918)  36.6 (1,857)  15.9 (809)  84 |
| **Ever use of hormonal replacement therapy, n (%)**  Yes  No  *Missing (n)* | 44.8 (12,631)  55.2 (15,546)  543 | 46.8 (2,155)  53.2 (2,446)  91 | 46.5 (1,973)  53.5 (2,269)  81 | 44.8 (6,386)  55.2 (7,881)  279 | 41.8 (2,117)  58.2 (2,950)  92 |
| **Ever breastfed, n (%)**  Yes  No  *Missing (n)* | 82.1 (23,235)  17.9 (5,060)  425 | 84.3 (3,898)  15.7 (725)  69 | 76.7 (3,276)  23.3 (995)  52 | 82.7 (11,868)  17.3 (2,474)  204 | 82.9 (4,193)  17.1 (866)  100 |
| Note: N/n: Numbers; p: Percentile; %: Percentage; BMI: Body Mass Index; y: Years; g/d: gram per day. ^1^According to the general data protection regulation, no numbers below 5 is displayed, *Numbers are rounded to the first decimal place. | | | | | |

# Supplementary Table 7: Baseline socio-demographic, lifestyle, and reproductive characteristics of postmenopausal women in the Diet, Cancer, and Health cohort and of those who were diagnosed with first primary malignant breast cancer during follow-up, separately (complete cases)

| **Characteristics^1^*** | **Total cohort** (N= 24,543) | **Cases** (n= 1,591) |
| --- | --- | --- |
| **Age at baseline (median, p10-p90)** | 56.8 (51.5-63.4) | 56.7 (51.5, 63.2) |
| **Educational level, % (n)**  Primary  Technical/professional school  Secondary school  Higher education | 32.3 (7,938)  46.3 (11,362)  11.4 (2,802)  9.9 (2,441) | 30.4 (483)  46.1 (733)  11.6 (185)  11.9 (190) |
| **BMI, % (n)**  <18.5 kg/m^2^  18.5-24.9 kg/m^2^  25-<30 kg/m^2^  ≥30 kg/m^2^ | 1.20 (292)  50.2 (12,311)  34.7 (8,514)  14.0 (3,426) | 0.8 (13)  51.1 (813)  33.4 (531)  14.7 (234) |
| **Median lifetime intake of alcohol (g/d), (median, p10-p90)^2^** | 6.6 (0.8-18.8) | 7.3 (1.1-20.6) |
| **Smoking status, % (n)**  Never  Former  Smoker | 43.0 (10,562)  24.9 (6,118)  32.0 (7,863) | 44.1 (701)  24.2 (385)  31.7 (505) |
| **Physical activity level, % (n)**  Inactive  Moderately inactive  Moderately active  Active | 10.7 (2,632)  32.6 (7,994)  24.7 (6,067)  32.0 (7,850) | 10.1 (160)  33.3 (530)  26.0 (413)  30.7 (488) |
| **Age at menarche, % (n)**  ≤12  13-14  >14 | 23.4 (5,741)  49.5 (12,138)  27.2 (6,664) | 25.5 (405)  48.3 (769)  26.2 (417) |
| **Age at first full term pregnancy, % (n)**  No full-term pregnancy  ≤21y  22-26y  >26y | 11,9 (2,917)  29.6 (7,268)  38,9 (9,546)  19,6 (4,812) | 14.8 (236)  27.4 (436)  36.4 (579)  21.4 (340) |
| **Ever use of hormonal replacement therapy, % (n)**  Yes  No | 47.9 (11,747)  52.1 (12,796) | 60.1 (956)  39.9 (635) |
| **Ever breast fed, % (n)**  Yes  No | 82.0 (20,132)  18.0 (4,411) | 79.4 (1,263)  20.6 (328) |
| Note: N/n: Numbers; p: Percentile; %: Percentage; BMI: Body Mass Index; y: Years; g/d: gram per day. ^1^ Complete case data, ^2^Information only available for 24,456 women and 1,587 cases of breast cancer, respectively, *Numbers are rounded to the first decimal place. | | |

# References

1. Proust-Lima C, Saulnier T, Philipps V, et al (2023) Describing complex disease progression using joint latent class models for multivariate longitudinal markers and clinical endpoints. Stat Med 42:3996–4014. https://doi.org/10.1002/sim.9844

2. Lennon H, Kelly S, Sperrin M, et al (2018) Framework to construct and interpret latent class trajectory modelling. BMJ Open 8:e020683. https://doi.org/10.1136/bmjopen-2017-020683

3. Textor J, van der Zander B, Gilthorpe MS, et al (2016) Robust causal inference using directed acyclic graphs: the R package “dagitty”. Int J Epidemiol 45:1887–1894. https://doi.org/10.1093/ije/dyw341
